# Functional and structural characterization of *Stenotrophomonas maltophilia* EntB, an unusual form of isochorismatase for siderophore synthesis

**DOI:** 10.1107/S2053230X2500490X

**Published:** 2025-06-04

**Authors:** Megan Y. Nas, Jeffrey Gabell, Nicole Inniss, George Minasov, Ludmilla Shuvalova, Karla J. F. Satchell, Nicholas P. Cianciotto

**Affiliations:** a Department of Microbiology–Immunology Northwestern University Feinberg School of Medicine Chicago IL60611 USA; b Center for Structural Biology of Infectious Diseases Northwestern University Feinberg School of Medicine Chicago IL60611 USA; c Department of Pharmacology Northwestern University Feinberg School of Medicine Chicago IL60611 USA; Institut Pasteur de Montevideo, Uruguay

**Keywords:** *Stenotrophomonas maltophilia*, siderophores, enterobactin, isochorismatases, EntB, isochorismate lyases

## Abstract

Clinical and environmental isolates of *Stenotrophomonas maltophilia* produce an enterobactin-like siderophore that promotes bacterial growth under low-iron conditions. Although prior mutational and bioinformatic analyses indicated that most of the enzymes encoded by the *S. maltophilia entCEBB′FA* locus are suitably reminiscent of their counterparts in *Escherichia coli* and other bacteria, *Stenotrophomonas* EntB was unusual. In bacteria producing enterobactin-related molecules, EntB and its homologs are usually multi-domain proteins in which the amino portion acts as an isochorismatase and the carboxy domain serves as an aryl carrier protein (ArCP). However, in *S. maltophilia* the isochorismatase and ArCP functions are encoded by two distinct genes: *entB* and *entB′*, respectively. Current mutant analysis was used to first confirm that *S. maltophilia entB* is needed for siderophore activity and bacterial growth in iron-depleted media. A crystal structure of *S. maltophilia* EntB was then obtained. The structure aligned with the N-terminal portion of EntB from *E. coli* and VibB from *Vibrio cholerae*, affirming the protein to be a single-domain isochorismatase. However, *S. maltophilia* EntB also aligned with the single-domain PhzD from *Pseudomonas aeruginosa*, which is a key enzyme involved in the biosynthesis of the antimicrobial compound phenazine. *BLASTP* searches indicated that *entB* and its neighboring genes are fully conserved amongst *S. maltophilia* strains but are variably present in other *Stenotrophomonas* species. The closest homologs to *S. maltophilia* EntB outside the genus were hypothetical proteins/putative isochorismatases in some Gram-negative bacteria (for example *Pseudomonas* spp. and *Xanthomonas* spp.), Gram-positive bacteria (*Streptomyces* spp. and *Bacillus subtilis*) and fungi (for example *Rhizopus arrhizus* and *Knufia peltigerae*).

## Abstract 2

The crystal structure of *S. maltophilia* EntB revealed an unusual form of isochorismate lyase that is involved in siderophore biosynthesis.

## 1. Introduction

Besides being common in water, soil and plant material, *Stenotrophomonas maltophilia* is an increasingly important human pathogen that causes pneumonia, bacteremia and other infections (Brooke, 2021 ▸). *S. maltophilia* is particularly concerning in cystic fibrosis patients (Terlizzi *et al.*, 2023 ▸). Many *S. maltophilia* strains are resistant to a range of antibiotics, making *S. maltophilia* infections difficult to treat (Kunz Coyne *et al.*, 2023 ▸). Among other things, *S. maltophilia* utilizes its flagella, pili, lipopolysaccharide, secretion systems, biofilm formation and iron acquisition to survive in the environment and in human hosts (Bhaumik *et al.*, 2023 ▸; Mikhailovich *et al.*, 2024 ▸; DuMont & Cianciotto, 2017 ▸; Cobe *et al.*, 2024 ▸; Di Bonaventura *et al.*, 2023 ▸; Crisan *et al.*, 2024 ▸). *S. maltophilia* uses various pathways to acquire iron, including the use of siderophores for ferric iron uptake, membrane transporters for the assimilation of ferrous iron, and heme/hemin uptake (Mikhailovich *et al.*, 2024 ▸; Pan *et al.*, 2022 ▸; Liao *et al.*, 2022 ▸; Shih *et al.*, 2022 ▸; Yeh *et al.*, 2025 ▸).

As a follow-up to early genomic analyses (Adamek *et al.*, 2014 ▸; García *et al.*, 2015 ▸), we previously reported that the *S. maltophilia* chromosome has (i) a locus that includes six open reading frames (ORF) that are predicted to encode enzymes for the synthesis of an enterobactin-related, catecholate-type siderophore (*entCEBB′FA*) and (ii) other genes that are predicted to mediate the export and import of a siderophore (Nas & Cianciotto, 2017 ▸). Compatible with these genomic data, many strains of *S. maltophilia*, including the often-studied clinical isolate K279a, secrete a siderophore activity that is detected by the Chrome Azurol S (CAS), Arnow or Rioux assays (Nas & Cianciotto, 2017 ▸; Berg *et al.*, 1996 ▸; Minkwitz & Berg, 2001 ▸; Garcia *et al.*, 2012 ▸; Peralta *et al.*, 2012 ▸; Williams *et al.*, 2012 ▸; Singh & Jha, 2017 ▸; Kalidasan *et al.*, 2018 ▸; Alcaraz *et al.*, 2018 ▸; Liao *et al.*, 2020 ▸; Hisatomi *et al.*, 2021 ▸). Based on the results of further biochemical tests and bioassays, the siderophore activity of strain K279a proved not to be enterobactin but rather consisted of the catecholate 2,3-dihydroxybenzoylserine and likely other enterobactin-related molecules (Nas & Cianciotto, 2017 ▸; Hisatomi *et al.*, 2021 ▸). By documenting the loss of CAS, Arnow and Rioux activity in an *entC* mutant of strain K279a, we determined that genes in the *ent* locus are required for production of the *S. maltophilia* siderophore (Nas & Cianciotto, 2017 ▸). Being quite similar in amino-acid sequence to the known EntC proteins from other bacteria (Nas & Cianciotto, 2017 ▸), *S. maltophilia* EntC is likely to mediate the first step in siderophore synthesis, *i.e.* the conversion of chorismic acid to isochorismate (Reitz *et al.*, 2017 ▸; Ouellette *et al.*, 2022 ▸). Subsequent mutagenesis of *S. maltophilia* strain kJ demonstrated an additional requirement for *entA* and *entF* in synthesis of the siderophore activity (Liao *et al.*, 2020 ▸; Wu, Chen *et al.*, 2022 ▸). Whereas the *S. maltophilia ent* genes are expressed upon bacterial growth under low-iron conditions, they are repressed by the ferric uptake regulator (Fur) protein during growth under high-iron conditions, as is the case for many other bacterial genes involved in iron assimilation (Nas & Cianciotto, 2017 ▸; Liao *et al.*, 2020 ▸; Wu, Li *et al.*, 2022 ▸). Finally, further transcriptomic analysis revealed that the *ent* genes exhibit increased expression when *S. maltophilia* exists in a biofilm (Wicaksono *et al.*, 2022 ▸). We now report that the *S. maltophilia* EntB protein is also required for siderophore synthesis and bacterial growth under low-iron conditions. A crystal structure determination revealed EntB to be an unusual, single-domain form of isochorismatase enzyme.

## 2. Materials and methods

### 2.1. Bacterial strains and standard media

The clinical isolate *S. maltophilia* K279a (American Type Culture Collection strain BAA-2423) was used as both our wild-type strain and the parental control for the *entB* mutant (below; DuMont & Cianciotto, 2017 ▸; Nas & Cianciotto, 2017 ▸; Cobe *et al.*, 2024 ▸). The *entC* mutant of K279a used in this study (*i.e.* strain NUS8) has previously been described (Nas & Cianciotto, 2017 ▸). *S. maltophilia* wild type and mutants were routinely cultured at 37°C on Luria–Bertani (LB) agar or in LB broth. *Escherichia coli* strain DH5α (Life Technologies) served as a host strain for the cloning and propagation of recombinant plasmids and was maintained on LB agar or in LB broth.

### 2.2. Mutant construction

An *entB* mutant of strain K279a (*i.e.* NUS28) with a deletion of the entire *entB*-coding region (SMLT_RS13410) was obtained using a PCR overlap extension protocol as described previously (DuMont *et al.*, 2015 ▸). *S. maltophilia* DNA was isolated as before (Karaba *et al.*, 2013 ▸). The primers used for sequencing and PCR (from Integrated DNA Technologies) are listed in Supplementary Table S1. To begin, the 5′ and 3′ regions that flank *entB* were PCR-amplified from K279a DNA with Platinum Taq polymerase (Life Technologies) using the primer pair MN78 and MN79 and the primer pair MN80 and MN81, respectively. A Flp recombination target (FRT)-flanked chloramphenicol cassette was PCR-amplified from pKD3 using primers MN82 and MN83 (Nas & Cianciotto, 2017 ▸). The overlap extension PCR mixture contained 60 ng each of the three previous PCR products. Three cycles of PCR were performed using HiFi polymerase before the addition of primers MN78 and MN81 for 30 more cycles. A band corresponding to the correct size (∼1.8 kb) was gel-purified, digested with EcoRI and HindIII (New England BioLabs) and ligated into pEX18Tc digested with the same enzymes, yielding pEXΔ*entB*::*frt-cat-frt*. The new plasmid was introduced into *E. coli* S17-1 and then mobilized into *S. maltophilia* K279a by conjugation. Transconjugants were plated onto LB agar supplemented with tetracycline, chloramphenicol and norfloxacin. Since strain K279a is resistant to norfloxacin (Karaba *et al.*, 2013 ▸), the inclusion of this antibiotic selects against the outgrowth of *E. coli* S17-1. Resistant colonies were plated onto LB agar containing sucrose and chloramphenicol to select for cells in which recombination and loss of pEX18Tc has occurred.

### 2.3. Genome searches

*BLASTP* at the NCBI was used to search the genome database for *Stenotrophomonas* and non-*Stenotrophomonas* proteins with primary-sequence similarity to EntB and the other Ent proteins of *S. maltophilia* K279a (Boratyn *et al.*, 2013 ▸).

### 2.4. Bacterial growth in low-iron media and siderophore assays

*S. maltophilia* growth under varying levels of iron limitation was performed as before (Nas & Cianciotto, 2017 ▸). Briefly, after overnight incubation on LB agar at 37°C, colonies of wild-type or mutant bacteria were inoculated into LB broth and incubated for 16 h with shaking. Bacteria from these cultures were then inoculated into 50 ml Stainer–Scholte minimal medium containing casamino acids (SSC) that was depleted of free iron by the addition of 125, 150, 175 or 200 µ*M* of the ferrous iron chelator 2,2′-dipyridyl (DIP). As before (Nas & Cianciotto, 2017 ▸), the SSC base consists of, per litre, 240 mg l-proline, 670 mg l-glutamic acid, 40 mg l-cystine, 2500 mg NaCl, 500 mg HK_2_PO_4_, 200 mg KCl, 100 mg MgCl_2_·6H_2_O, 20 mg CaCl_2_, 10 mg FeSO_4_·7H_2_O, 6075 mg Tris buffer, 20 mg ascorbic acid, 4 mg niacin, 100 mg glutathione and 0.1% casamino acids. The double-distilled water used to make the medium was deferrated by passage through a column packed with Chelex-100 beads. The cell suspensions were incubated at 37°C with shaking, and growth was monitored every 12 h for the next 48 h by obtaining the optical density of the samples at 600 nm (OD_600_). Siderophore production by *S. maltophilia* was ascertained as before (Nas & Cianciotto, 2017 ▸). Briefly, at the indicated time point, OD_600_-normalized low-iron (DIP-containing) SSC broth cultures (above) were centrifuged and the resultant supernatants were sterilized by passage through 0.22 µm syringe filters (EMD Millipore). The cell-free supernatants were then tested, as before, for the presence of siderophore using the Rioux assay (Nas & Cianciotto, 2017 ▸; Payne, 1994 ▸; Rioux *et al.*, 1983 ▸). The levels of siderophore reactivity produced by *S. maltophilia* strains were expressed as 2,3-dihydroxybenzoic acid (DHBA) equivalents, as calculated from a standard curve generated using a range of concentrations of purified DHBA.

### 2.5. Cloning, expression, purification and crystallization of *S. maltophilia* EntB

The *entB* gene from strain K279a (GenBank CAQ46281; codons 1–210) was cloned using ligation-independent cloning into the pMSCG53 vector (Eschenfeldt *et al.*, 2013 ▸), which encodes genes that provide tRNAs for rare codons and ampicillin resistance; EntB is expressed as an N-terminally, 6×His-tagged fusion protein that contains a Tobacco etch virus (TEV) protease cleavage site for tag removal. The resulting plasmid was transformed into *E. coli* BL21 (DE3) (Magic) cells (Kwon & Peterson, 2014 ▸). The starting overnight culture was grown in LB broth supplemented with 130 µg ml^−1^ ampicillin and 50 ml^−1^ kanamycin at a temperature of 37°C with rotation at 220 rev min^−1^. The next day, 3 l of M9 medium (High Yield M9 selenomethionine medium, Medicilon Inc.) supplemented with 200 µg ml^−1^ ampicillin and 50 µg ml^−1^ kanamycin were inoculated with the overnight culture at 1:100 dilution and incubated at 37°C with rotation at 220 rev min^−1^ until the OD_600_ reached 1.8–2.0. Protein expression was induced using 0.6 m*M* isopropyl β-d-1-thiogalactopyranoside (IPTG) at 25°C and 220 rev min^−1^ for 14 h. The cells were harvested by centrifugation at 6000 rev min^−1^ for 10 min, resuspended (1 g of cells:5 ml lysis buffer) in lysis buffer (50 m*M* Tris pH 8.3, 0.5 *M* NaCl, 10% glycerol, 0.1% IGEPAL CA-630) and frozen at −30°C until purification. Frozen pellets were thawed and sonicated at 50% amplitude in 5 × 10 s cycles for 40 min in an ice bath. The lysate was clarified by centrifugation at 36 000*g* for 40 min at 4°C, the supernatant was collected and the protein was purified as described previously (Shuvalova, 2014 ▸). The purified EntB protein was concentrated to 8.2 mg ml^−1^ and then set up for crystallization at 8.0 mg ml^−1^ in buffer consisting of 10 m*M* Tris–HCl pH 8.3, 1 m*M* TCEP with and without 500 m*M* NaCl as 2 µl crystallization drops (1 µl protein:1 µl reservoir solution) in 96-well crystallization plates (Corning) using the commercial Classics II, PEGs II and ComPAS (Qiagen) crystallization screens. A diffraction-quality crystal of the protein grown from a condition with 3.0 *M* sodium formate (ComPAS condition No. 96) was cryoprotected in 4.0 *M* sodium formate and flash-cooled in liquid nitrogen for data collection.

### 2.6. Structure determination of *S. maltophilia* EntB

A data set was collected from the single crystal on beamline 21-ID-F of the Life Sciences Collaborative Access Team (LS-CAT) at the Advanced Photon Source, Argonne National Laboratory. Images were indexed, integrated and scaled using *HKL*-3000 (Minor *et al.*, 2006 ▸). Data-quality, structure-refinement and the final model statistics are shown in Table 1 ▸. The crystal belonged to the cubic space group *I*4_1_32 with one protein chain in the asymmetric unit. The structure was determined by the single anomalous dispersion (SAD) method using anomalous signal from the selenomethionine protein derivative. The initial model was built using the *HKL*-3000 structure-solution package and went through several rounds of refinement in *REFMAC* (Murshudov *et al.*, 2011 ▸). Manual model corrections were performed using *Coot* (Emsley & Cowtan, 2004 ▸). The water molecules were generated automatically using *ARP*/*wARP* (Morris *et al.*, 2003 ▸) followed by further rounds of refinement in *REFMAC*. Chloride and sodium ions and formate molecules were fitted into electron-density maps manually and the structure was further refined in *REFMAC* using translation–libration–screw (TLS) group corrections, which were made by the *TLS Motion Determination* (*TLSMD*) server (Painter & Merritt, 2006 ▸). The quality of the model during refinement and final validation of the structure was performed using *MolProbity*(Chen *et al.*, 2010 ▸; http://molprobity.biochem.duke.edu/). The *Sm*EntB structure was deposited in the RCSB PDB (https://www.rcsb.org/) with PDB code 7l6j. *ESPript* (Robert & Gouet, 2014 ▸) was used to generate alignments with homologs from *P. aeruginosa*, *Streptomyces* sp. ATCC 700974, *V. cholerae* and *E. coli*. The electrostatic surface of *Sm*EntB was predicted using *APBS *(Jurrus *et al.*, 2018 ▸) in *PyMOL* (Schrödinger).

## 3. Results

### 3.1. *S. maltophilia entB* promotes siderophore production and bacterial growth in iron-depleted media

**Figure 1:**
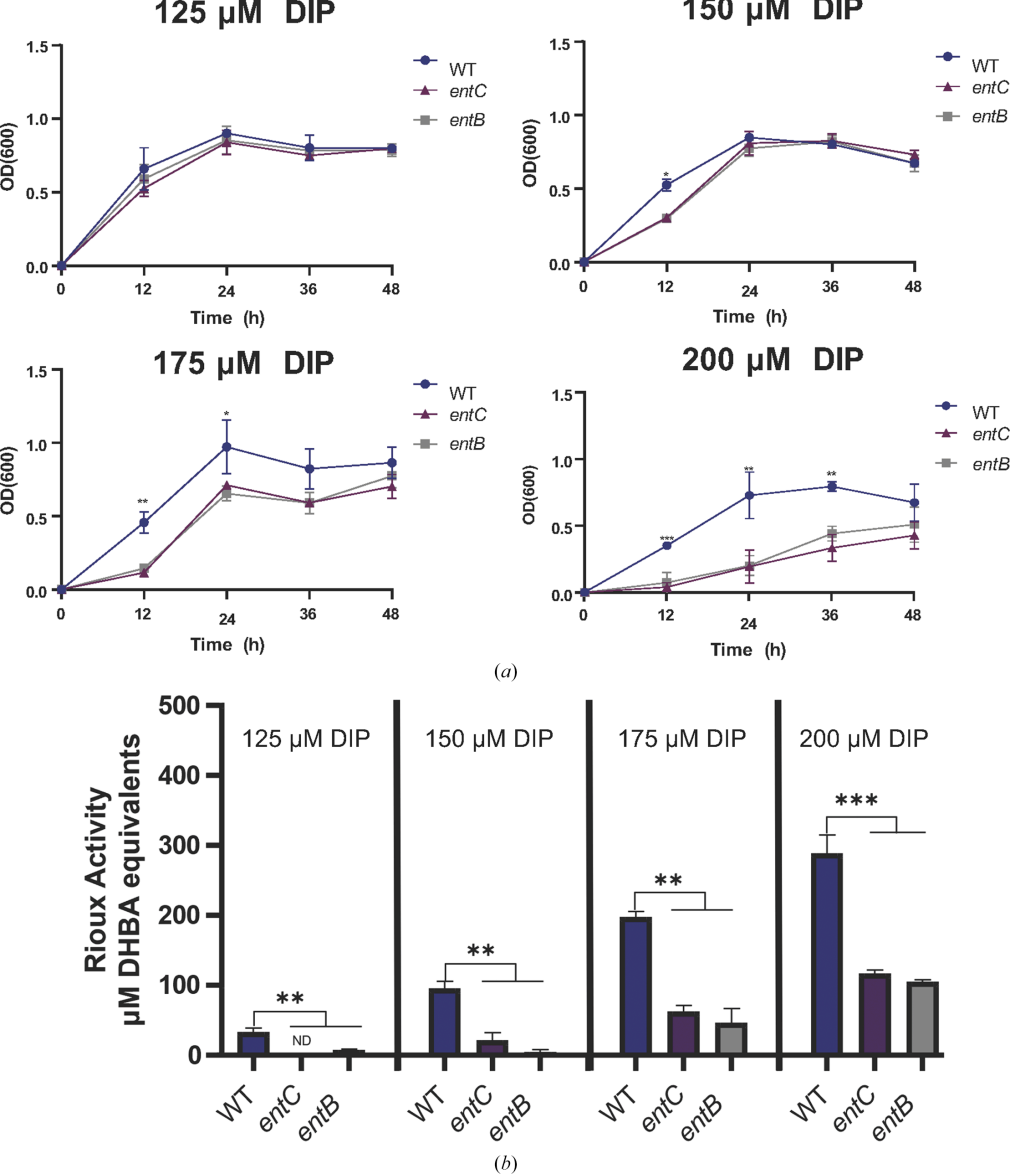
Effect of *entB* on *S. maltophilia* growth and siderophore production. Strain K279a (WT), the *entC* mutant NUS8 (*entC*) and the *entB* mutant NUS28 (*entB*) were inoculated into SSC medium containing 125, 150, 175 or 200 µ*M* DIP and incubated at 37°C for 48 h. Bacterial growth was monitored spectrophotometrically (*a*). At 36 h, the culture supernatants were assessed for levels of reactivity in the Rioux assay, measured as net 2,3-dihydroxybenzoic acid (DHBA) equivalents (*b*). Asterisks indicate significant differences between the WT and mutant strains (Student’s *t*-test; **, *p* < 0.005; ***, *p* < 0.001). Data are presented as the means and standard deviations of results from three independent experiments (*n* = 3 each). ‘ND’ indicates undetectable Rioux activity.

In *E. coli* and in most other producers of enterobactin-type siderophores, EntB and its homologs are multi-domain proteins in which (i) their amino portion acts as an isochorismatase to produce the siderophore intermediate 2,3-di­hydro-2,3-dihydroxybenzoate, which is then acted on by EntA to make 2,3-dihydroxybenzoic acid (DHBA), and (ii) their carboxy domain serves as an aryl carrier protein (ArCP) to then aid EntE, EntF and EntD in linking DHBA to serine (Reitz *et al.*, 2017 ▸; Bin & Pawelek, 2024 ▸; Conley *et al.*, 2024 ▸). However, in *S. maltophilia*, the isochorismatase and ArCP functions are encoded by two separate ORFs. In strain K279a, the isochorismatase domain is encoded by ORF RS13410 (alternate tag smlt2820), which has been referred to as *entB*or *entB1*, and the ArCP domain is encoded by ORF RS13405 (smlt2819), which has been denoted as *entB′*, *entB2* or *entD* (Supplementary Fig. S1; Nas & Cianciotto, 2017 ▸; Liao *et al.*, 2020 ▸). This unusual arrangement is reminiscent of what occurs in *Streptomyces* species that make griseobactin, where EntB is like *Streptomyces* DhbB and EntB′ is akin to *Streptomyces* DhbG (Reitz *et al.*, 2017 ▸; Patzer & Braun, 2010 ▸; Albright *et al.*, 2014 ▸). To test the importance of EntB in siderophore synthesis by *S. maltophilia*, we generated an *entB*-deletion mutant of strain K279a and then tested the culture supernatant of the mutant for siderophore activity, as before (Nas & Cianciotto, 2017 ▸). Like the previously described *entC* mutant (Nas & Cianciotto, 2017 ▸), the *entB* mutant grew similarly to the parental wild-type strain K279a when inoculated into SSC medium that was made iron-limiting by the inclusion of 125 µ*M* DIP (Fig. 1 ▸*a*). However, when we assayed culture supernatants for siderophore activity, the *entB* mutant, like the *entC* mutant (Nas & Cianciotto, 2017 ▸), showed reduced reactivity in the Rioux assay (Fig. 1 ▸*b*). To further document the importance of EntB in *S. maltophilia*, we tested the relative ability of the *entB* mutant to grow in media that were increasingly limited in iron availability. Whereas the *entB* mutant grew almost nearly as well as the wild type did in medium containing 150 µ*M* DIP, its growth was more notably impaired in media containing 175 or 200 µ*M* DIP (Fig. 1 ▸*a*). The *entB* mutant continued to display a loss of siderophore when cultured in media containing higher levels of DIP (Fig. 1 ▸*b*). Currently, the basis for the residual siderophore activity in the supernatants of the *entB* and *entC* mutants is not clear, since *S. maltophilia* does not encode homologs or isoenzymes of EntB or EntC, nor are there reports of the bacterium encoding or secreting another type of catecholate siderophore or molecule that is reactive in the Rioux assay (Nas & Cianciotto, 2017 ▸; Liao *et al.*, 2020 ▸; Hisatomi *et al.*, 2021 ▸; Yeh *et al.*, 2025 ▸). Overall, these data indicated that *entB* is required for *S. maltophilia* siderophore activity and bacterial growth under iron-limiting conditions.

### 3.2. EntB homologs in *S. maltophilia* strains, other *Stenotrophomonas* species and non-*Stenotrophomonas* genera

*BLASTP* searches revealed that EntB homologs are present in 41 of 41 other sequenced strains of *S. maltophilia* examined, with these proteins sharing 98% amino-acid identity with the K279a prototype (Supplementary Table S2). The other *ent* genes were similarly conserved (Supplementary Table S2). These data also indicated that the apparent separation of the isochorismatase and ArCP functions is not an oddity of strains K279a and kJ but is a conserved feature of the species. From further searches, homologs to EntB and all of the other *ent* gene products were identified in *S. indicatrix*, *S. lactitubi*, *S. pavanii*, *S. bentonitica* and *S. rhizophila* (Supplementary Table S3). EntB, as well as EntA, EntF, EntE and EntC, but not EntB′, was also detected in *S. muris* and *S. riyadhensis* (Supplementary Table S3). When comparing with genomes outside *Stenotrophomonas*, K279a EntB had high similarity (*i.e.* 68–99% amino-acid identity; *E* < 7 × 10^−92^) to hypothetical isochorismatases from a broad range of Gram-negative bacteria, Gram-positive bacteria and fungi. Supplementary Table S4 lists those hypothetical isochorismatases that had the greatest similarity to K279a EntB. However, *S. maltophilia* EntB also had 46% amino-acid identity (*E* = 1 × 10^−62^) to the characterized DhbB protein from *Streptomyces* spp., which, as noted above, is an example of a bacterium in which the isochorismatase and ArCP functions are encoded by two separate ORFs (Reitz *et al.*, 2017 ▸; Supplementary Table S4). *S. maltophilia* EntB also had predicted similarity to a siderophore-synthesis enzyme (VibB) from *V. cholerae* as well as a phenazine-synthesis enzyme (PhzD) from *P. aeruginosa* (see below).

### 3.3. Structural characterization of *S. maltophilia* EntB

**Figure 2:**
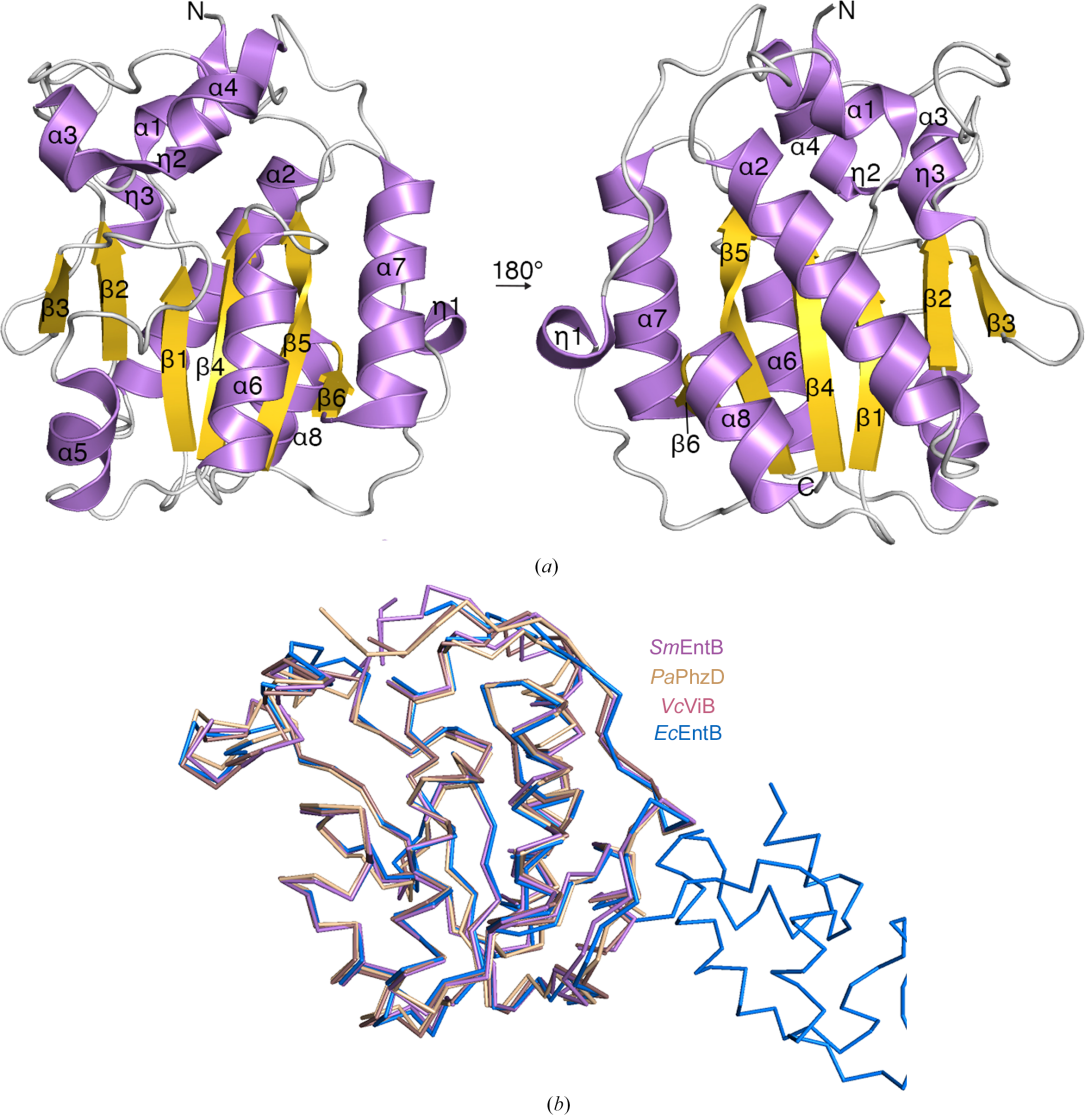
Structure of the *S. maltophilia* EntB protein. (*a*) The overall structure of *S. maltophilia* EntB, depicted as a cartoon. Secondary-structural elements are labeled and α-helices are colored lilac, β-strands gold and loops light gray. The image on the left was rotated 180° towards the viewer, resulting in the image on the right. The N- and C-termini are labeled. (*b*) Structural overlay of the carbon backbones of *S. maltophilia* EntB (*Sm*EntB; PDB entry 7l6j, lilac), *P. aeruginosa* PhzD (*Pa*PhzD; PDB entry 1nf9, wheat), *V. cholerae* VibB (*Vc*VibB; PDB entry 3tg2, light brown) and *E. coli* EntB (*Ec*EntB; PDB entry 2fq1, blue).

The apparent separation of isochorismatase and ArCP function in *S. maltophilia* prompted us to discern the structure of EntB from K279a to identify potentially key structural differences across the known isochorismatase domains. The *S. maltophilia* EntB (*Sm*EntB) structure was determined at 1.78 Å resolution. Structure statistics are listed in Table 1 ▸. Overall, the *Sm*EntB structure consists of a single globular domain which is comprised of mixed α/β elements (Fig. 2 ▸*a*). The protein core consists of a six-stranded parallel β-sheet (β3–β2–β1–β4–β5–β6). Helices η1, α5, α6, α7, α2, α8 and η3 surround and cover most residues of the core β-sheet from the solvent (Fig. 2 ▸*a*). The helices α1, α3, η2 and α4 are separated from the central core and form a lobe that sits on top of the protein core (Fig. 2 ▸*a*). The cleft that is located between the lobe and the central core of the protein is part of the putative active site. Although a single chain is observed in the asymmetric unit, crystallographic symmetry revealed the presence of a dimer. Analytical size-exclusion chromatography estimated a molecular weight of 61.8 kDa, consistent with dimerization (Supplementary Fig. S2).

A *DALI* search (Holm, 2022 ▸) using the coordinates of the *Sm*EntB model identified multiple isochorismatase domain-containing proteins with high structural similarity, including *E. coli* EntB, which, as noted above, is involved in the synthesis of enterobactin (*Ec*EntB; 45% identity), *V. cholerae* VibB, which promotes synthesis of the enterobactin-related siderophore vibriobactin (*Vc*VibB; 45% identity), and *P. aeruginosa* PhzD, which helps to synthesize the antimicrobial compound phenazine (*Pa*PhzD; 46% identity) (Drake *et al.*, 2006 ▸; Liu *et al.*, 2012 ▸; Parsons *et al.*, 2003 ▸). When the asymmetric unit chain was overlaid with these structures (Fig. 2 ▸*b*), *Sm*EntB had an average root-mean-square deviation (r.m.s.d.) of 1.10 Å across main-chain C^α^ atoms. The most obvious differences between these structures are the presence of the C-terminal ArCP domain in *Ec*EntB. *Vc*VibB also has a fused C-terminal ArCP domain, but this was not resolved in the structure (Drake *et al.*, 2006 ▸; Liu *et al.*, 2012 ▸). However, like *Sm*EntB, *Pa*PhzD is an independent isochorismatase and it catalyzes the conversion of 2-amino-2-deoxyisochorismate (ADIC) to *trans*-2,3-dihydro-3-hydroxyanthranilate and related vinyl ethers utilized in phenazine biosynthesis (Parsons *et al.*, 2003 ▸). The overall structural similarity between *Sm*EntB and *Pa*PhzD with the N-termini of *Vc*VibB and *Ec*EntB supports our hypothesis that *Sm*EntB is an isochorismatase involved in siderophore synthesis.

**Figure 3:**
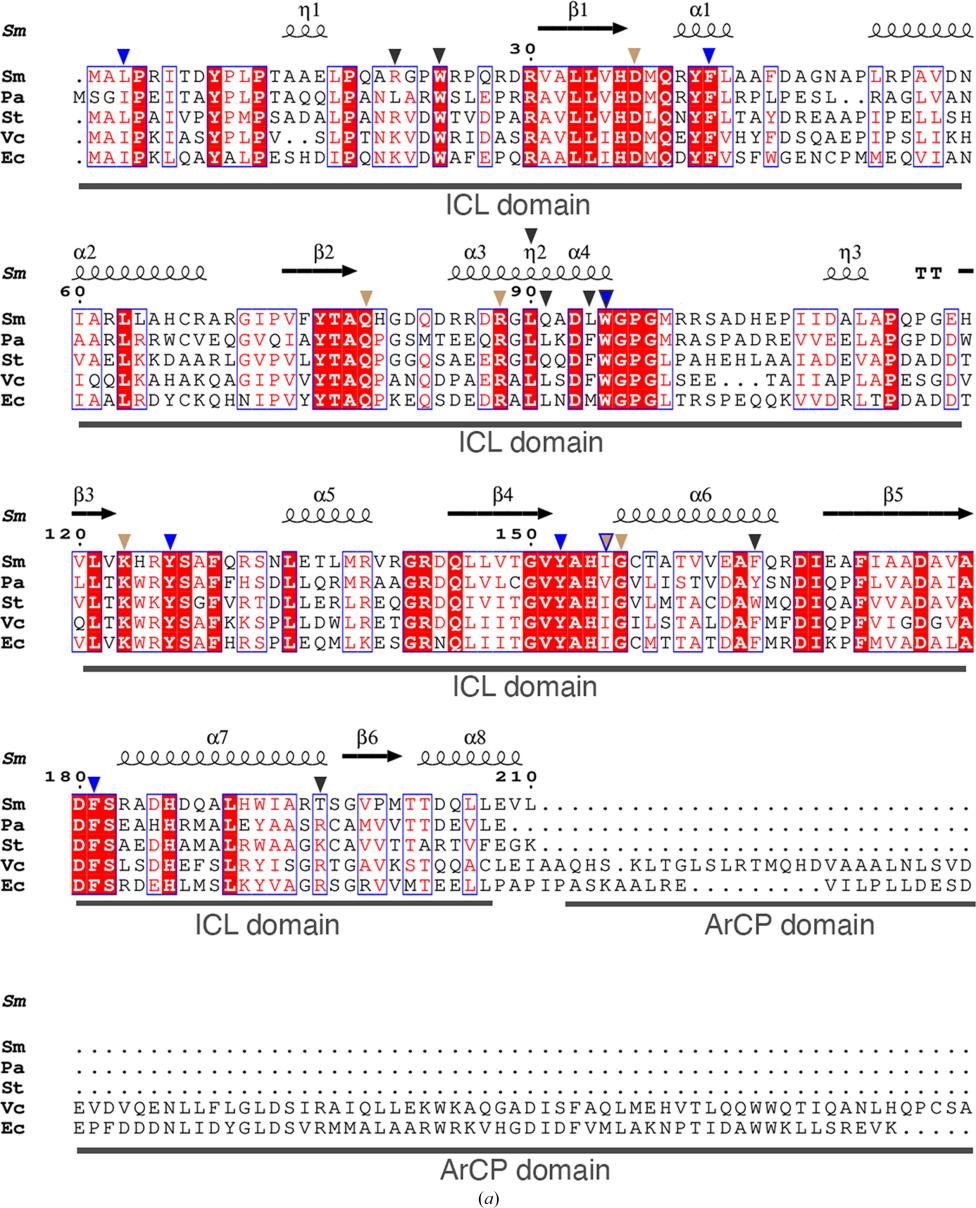

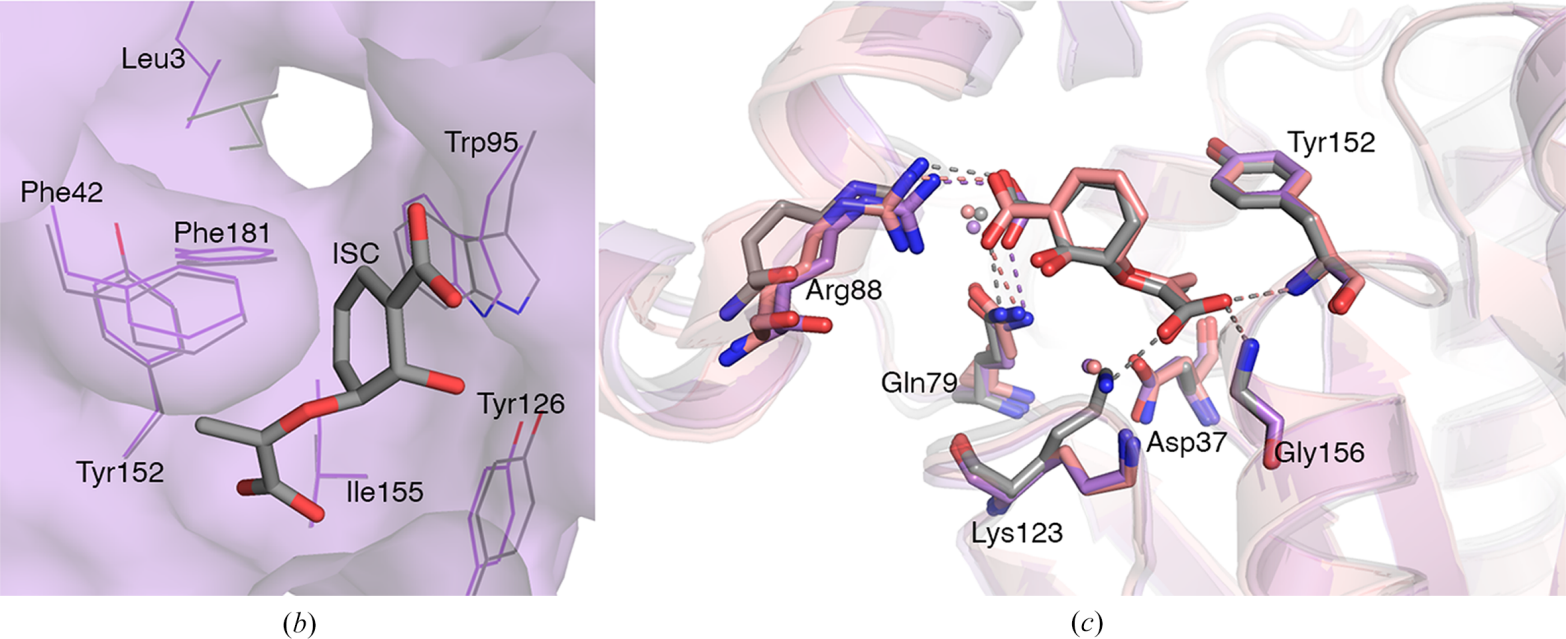
Structure–sequence alignments of EntB homologs. (*a*) *ESPript* was used to generate an alignment of EntB homologs from *S. maltophilia* (Sm), *P. aeruginosa* (Pa), *Streptomyces* sp. ATCC 700974 (St), *V. cholerae* (Vc) and *E. coli* (Ec). The isochorismatase (ICL) and ArCP domains of the enzymes are marked below the sequences. The secondary-structural elements from *Sm*EntB are depicted above the sequences. Inverted blue triangles mark residues of the hydrophobic pocket, inverted tan triangles mark those that interact with isochorismate (ISC) in the *Pa*PhzD and *Vc*VibB structures, and inverted gray triangles mark residues of the dimer interface that align across all homologs. An inverted tan triangle outlined in blue marks a single residue that is characterized as being part of the hydrophobic pocket and interacts with ISC, and an inverted gray triangle outlined in blue marks a residue that is predicted to interact with ISC and contribute to the dimer interface. (*b*) An overlay of a surface projection of *Sm*EntB and *Pa*PhzD–ISC depicting an enlarged view of the ISC binding pocket. Residues that are conserved in this pocket are depicted as lines with their carbon backbones colored lilac for *Sm*EntB and gray for *Pa*PhzD, N atoms in blue and O atoms in red. Residues are numbered according to the *Sm*EntB structure. (*c*) An overlay of the *Sm*EntB (lilac), *Pa*PhzD (PDB entry 1nf8, gray) and *Vc*VibB (salmon) structures depicted as transparent cartoons. An enlarged view of the active site is shown. ISC from *Pa*PhzD (gray C atoms) and *Vc*VibB (salmon C atoms) and a formate molecule (lilac C atoms) are shown. Residues that interact with ISC or formate and are conserved in all three structures are depicted as sticks, with N and O atoms colored as in (*b*). Water molecules identified in at least two of the active sites of the structure are shown as small spheres colored according to the carbon backbones. Dashed lines indicate hydrogen bonds and are colored according to the carbon backbones. Residues are numbered according to the *Sm*EntB structure.

In the reported crystal structures of *Pa*PhzD (PDB entry 1nf9) and *Vc*VibB (PDB entry 3tg2), the isochorismatase domains were captured in complex with isochorismate. An overlay of the *Sm*EntB and *Pa*PhzD–isochorismate structures (Supplementary Fig. S3) revealed that the residues of the active site of *Sm*EntB and *Pa*PhzD aligned very well, and that the ligand bound to the *Pa*PhzD active site would fit in a deep pocket previously characterized as being comprised of mostly hydrophobic residues (Parsons *et al.*, 2003 ▸). Mapping the electrostatic potentials on the surface of *Sm*EntB suggested that this putative isochorismate-binding site is mostly hydrophobic, but it is adjacent to charged residues (Supplementary Fig. S3). The hydrophobic residues of the pocket (Leu3, Phe42, Trp95, Tyr126, Tyr1552, Ile155 and Phe181) are conserved in *Sm*EntB and other structural homologs (Figs. 3 ▸*a* and 3 ▸*b*), suggesting that isochorismate or other vinyl ethers may fit into the active site of *Sm*EntB. As observed in *Pa*PhzD, the putative *Sm*EntB active site is capped by residues 79–104 and the N-terminus, which in our structure is comprised of α1, α3, η2 and α4.

A comparison of the active sites of all three enzymes (Fig. 3 ▸*c*) demonstrated that key residues involved in isochorismate binding and hydrolysis are conserved. The side chains of the conserved residues Gln79 and Arg88 are positioned similarly in all three enzymes and stabilize the ring carboxylate group of the substrate in *Pa*PhzD and *Vc*VibB. In our structure, Gln79 and Arg88 interact with a formate, which occupies the same position as the ring carboxylate. The O1′ atom of the pyruvyl carboxylate is hydrogen-bonded to the main-chain amides of Tyr152 and Gly156 in both *Pa*PhzD and *Vc*VibB, and these residues are also conserved. Although also conserved, the position of Lys123 differs between the three enzymes: in *Pa*PhzD the lysine side chain forms a hydrogen bond to the O2′ atom of the pyruvyl moiety, while in *Vc*VibB the lysine side chain is 3.7 Å away from the pyruvyl O2′ atom and the *Sm*EntB side chain is in the same position. Molecular dynamics of the *Vc*VibB complex suggest that a water atom might contribute to substrate hydrolysis by acting as a general acid to protonate the catalytic Asp37 residue prior to modification of isochorismate (Liu *et al.*, 2012 ▸). Indeed, in *Sm*EntB a water molecule is present in the same position, suggesting that the mechanism of substrate hydrolysis might be consistent with that proposed for *Vc*VibB. Lastly, all these proteins were confirmed as dimers in solution (Drake *et al.*, 2006 ▸; Liu *et al.*, 2012 ▸; Parsons *et al.*, 2003 ▸). The residues at the dimer interface are conserved overall across species, including Trp95, which contributes to the formation of the hydrophobic pocket and interacts with ISC in *Pa*PhzD (Fig. 3 ▸*a* and Supplementary Fig. S2).

## 4. Discussion

Analysis of the *Sm*EntB structure, along with mutant analysis of *entB* and other genes in the *ent* locus, indicates that *Sm*EntB is a key component of siderophore biosynthesis in *S. maltophilia*. Structural and sequence alignments demonstrate that *Sm*EntB shares sequence and structural similarity with (i) isochorismatase enzymes that are involved in siderophore production by *E. coli* (enterobactin) and *V. cholerae* (vibriobactin) and (ii) an isochorismatase enzyme that contributes to phenazine synthesis by *P. aeruginosa*. Interestingly, both *Sm*EntB and *Pa*PhzD are single-domain isochorismatase enzymes. Although the *Pa*PhzD structure was solved in complex with isochorismate, *Pa*PhzD acts on ADIC, iso­chorismate and chorismate, albeit with different efficiencies (Mavrodi *et al.*, 2001 ▸; Parsons *et al.*, 2003 ▸). These compounds or their related derivatives are intermediates in the synthesis of pyochelin, a different type of catecholate siderophore (Jeong *et al.*, 2024 ▸). However, to our knowledge, no direct link of *Pa*PhzD to siderophore synthesis in *P. aeruginosa* has been reported. Conversely, there is presently no evidence that *S. maltophilia* makes pyochelin or a phenazine (Pierson & Pierson, 2010 ▸; Huang *et al.*, 2024 ▸; Acharya *et al.*, 2024 ▸), although a recent isolate of the species appears to have a homolog of PhzF, which is another enzyme in the phenazine-synthesis pathway (Sharma *et al.*, 2024 ▸). On the other hand, the similarity of *Sm*EntB to *Vc*VibB suggests that there might be similarities between the *S. maltophilia* siderophore- and vibriobactin-synthesis pathways. A catalytic mechanism of isochorismate hydrolysis by *Vc*VibB has been proposed (Liu *et al.*, 2012 ▸), and a potentially conserved water in the catalytic site might suggest mechanistic similarity with *Sm*EntB. Yet, we cannot explain why *Sm*EntB evolved to be a single-domain enzyme whereas *Vc*VibB, like *Ec*EntB, has both isochorismatase and ArCP domains.

Current *BLASTP* searches indicated that *entB* and its neighboring genes are 100% conserved among clinical and environmental isolates of *S. maltophilia*, suggesting that the synthesis of a catecholate siderophore is quite important for the growth and survival of *S. maltophilia*. However, only about 25% of the other *Stenotrophomonas* species had the *entB*-containing operon, suggesting that another type of siderophore may be produced by other species in the genus. Putative isochorismatases with striking amino-acid identity to *Sm*EntB occurred in a broad range of microbes outside the *Stenotrophomonas* genus. This group included proteins from some species of *Pseudomonas* and *Xanthomonas*, a result that is not surprising given that *S. maltophilia* was once considered to be a member of the *Pseudomonas* and *Xanthomonas* genera (Brooke, 2021 ▸). However, the group of homologs also included representatives from a wide range of Gram-positive bacteria (for example *Bacillus subtilis* and *Streptomyces* sp.) and fungi (for example *Rhizopus arrhizus* and *Knufia peltigerae*). Thus, *Sm*EntB appears to be representative of a large but uncharacterized group of enzymes.

Given its role in pathogenesis, siderophore synthesis is considered to be a potential target for antimicrobial agents (Lamb, 2015 ▸). Indeed, chemical inhibitors of enterobactin and enterobactin-like synthesis pathways have been reported, including examples targeting species of *Acinetobacter*, *Mycobacterium*, *Pseudomonas* and *Yersinia* (Chi *et al.*, 2012 ▸; Vickery *et al.*, 2014 ▸; Foley *et al.*, 2014 ▸). Thus, the data reported here may help develop means for targeting the *S. maltophilia* pathogen.

## Supplementary Material

PDB reference: *S. maltophilia* EntB, 7l6j

Supplementary Tables and Figures. DOI: 10.1107/S2053230X2500490X/bus5002sup1.pdf

## Figures and Tables

**Table 1:** Data-quality and refinement statistics for the *S. maltophilia* EntB structure (PDB entry 7l6j) Values in parentheses are for the outer shell.

Data collection	
Space group	*I*4_1_32
*a*, *b*, *c* (Å)	171.69, 171.69, 171.69
α, β, γ (°)	90, 90, 90
Wavelength (Å)	0.97872
Resolution range (Å)	30.00–1.78 (1.81–1.78)
No. of unique reflections	41390 (2047)
*R*_merge_ (%)	11.1 (88.6)
*R*_p.i.m._ (%)	3.6 (28.7)
CC_1/2_ (%)	99.4 (85.1)
Completeness (%)	99.9 (100.0)
〈*I*/σ(*I*)〉	23.0 (3.3)
Multiplicity	10.3 (10.4)
Wilson *B* factor (Å^2^)	18.8
Refinement	
Resolution range (Å)	29.45–1.78 (1.83–1.78)
Completeness (%)	99.9 (99.9)
No. of reflections	
Working set	37260 (2872)
Free-*R* test set†	2031 (149)
*R*_work_/*R*_free_ (%)	13.7/15.4 (21.7/24.6)
Protein chains/atoms	1/1676
Ligand/solvent atoms	23/415
Mean temperature factor (Å^2^)	20.0
R.m.s.d., bond lengths (Å)	0.005
R.m.s.d., angles (°)	1.376
Ramachandran plot	
Favored (%)	98.0
Allowed (%)	1.0
Outside allowed (%)	1.0‡

† The free-*R* test-set size is 4.9%.

‡ Two residues, Ile155 and Ser197, which are outside the allowed region of the Ramachandran plot, are surrounded by well defined 2*F*_o_ − *F*_c_ electron density.
