# Supplementary material for: Functional and structural characterization of Stenotrophomonas maltophilia EntB, an unusual form of isochorismatase for siderophore synthesis
Source: Acta Crystallogr F Struct Biol Commun. 2025 Jun 4;81(Pt 7):287–96. doi: 10.1107/S2053230X2500490X (PMC12210187; doi:10.1107/S2053230X2500490X)
Supplement: Supplementary file 1 [file f-81-00287-sup1.pdf]

Acta Cryst F

STRUCTURAL BIOLOGY
COMMUNICATIONS

Volume 81 (2025)

Supporting information for article:

Functional and structural characterization of *Stenotrophomonas maltophilia* EntB, an unusual form of isochorismatase for siderophore synthesis

Megan Y. Nas, Jeffrey Gabell, Nicole Inniss, George Minasov, Ludmilla Shuvalova, Karla J. F. Satchell and Nicholas P. Cianciotto

Table S1 Primers used in this study

**Name**	**5' to 3' Sequence (Restriction sites underlined)**
MN78	AAAGAATTCGGATGACCCGGAGGAACTGATCGTGG
MN79	CGAAGCAGCTCCAGCCTACACACATCAGCGCGTTCCTCCTGTCTGTTGCAGATGACGCGCACGC
MN80	TAAGGAGGATATTCATATGTGATGGCTGCCGCGGGTGAGGTGCTGGATCTGGAGCGCATGC
MN81	AAAAAGCTTGGGTCGACCGGGCTCAGCCGGTCGG
MN82	GCGTGCGCGTCATCTGCAACAGACAGGAGGAACGCGCTGATGTGTGTAGGCTGGAGCTGCTTCG
MN83	GCATGCGCTCCAGATCCAGCACCTCACCCGCGGCAGCCATCACATATGAATATCCTCCTTA

Table S2 Presence of EntB and all other identified siderophore synthesis proteins in strains of *S. maltophilia*

Strain		Percent Identity to Siderophore Synthesis Proteins of *S. maltophilia* Strain K279a					
**Name**	**Source**	**EntA**	**EntF**	**EntB'**	**EntB**	**EntE**	**EntC**
K279a	clinical	100	100	100	100	100	100
R551-3	environmental	92	93	91	97	95	96
JV3	environmental	91	93	93	97	97	93
D457	unknown	94	94	93	98	96	91
ISMMS3	clinical	90	91	92	97	93	91
ISMMS2	clinical	93	96	96	99	97	96
ISMMS2R	clinical	93	96	96	99	97	96
AA1	environmental	87	86	86	94	92	79
OUC_Est10	environmental	91	94	94	98	98	96
AB550	environmental	94	95	94	98	96	90
FDAARGOS_325	clinical	100	99	96	99	99	99
CSM2	environmental	92	94	92	97	96	93
FDAARGOS_92	clinical	92	98	93	100	99	99
SJTH1	environmental	91	95	92	97	98	95
W18	environmental	94	95	98	100	98	96
SJTL3	environmental	93	93	92	97	96	95
FDAARGOS_507	clinical	94	97	93	100	99	99
X28	environmental	95	96	98	99	98	95
FDAARGOS_649	clinical	93	96	95	98	99	100
KMM 349	environmental	92	93	91	97	95	96
U5	environmental	84	83	85	93	93	81
Sm53	environmental	93	98	93	100	99	97
sm454	environmental	94	98	93	98	98	100
sm-RA9	unknown	92	92	91	97	95	96
SKK55	environmental	93	94	92	99	96	94
PEG-68	environmental	94	94	95	99	96	90
PEG-390	environmental	92	95	93	98	98	96
PEG-42	environmental	93	95	98	99	98	97
PEG-305	environmental	91	95	91	97	97	91
PEG-173	environmental	92	95	91	97	97	95

PEG-141	environmental	91	93	91	96	96	95
ICU331	environmental	96	98	93	100	100	100
CPBW01	environmental	92	95	95	99	97	96
MER1	environmental	85	83	85	92	92	82
NCTC10498	clinical	93	96	95	98	99	100
NEB515	environmental	98	97	93	100	99	97
CF13	clinical	94	98	93	100	99	99
NCTC10257	clinical	92	97	96	99	99	98
NCTC10258	clinical	98	98	93	99	99	98
NCTC10498	clinical	94	98	93	100	100	100
NCTC13014	environmental	94	95	94	99	98	97
NCTC10259	environmental	94	94	95	99	96	90
	**AVERAGE**	93	95	93	98	97	95

Table S3 EntB and other identified siderophore synthesis proteins in other *Stenotrophomonas* species

Homologs to EntB were not detected in the following Stenotrophomonas species: S. humi, S. nitritireducens, S. terrae, S. daejeonensis, S. chelatiphaga, S. acidaminiphila, S. ginsengisoli, S. koreensis, S. pictorum, S. panacihumi, S. dokdonensis, S. cyclobalanopsidis, S. tumulicola, S. pennii, and S. sepilia.

***Stenotrophomonas* Species and Strain**	**Percent Identity to Siderophore Synthesis Proteins of *S. maltophilia* Strain K279a**					
	**EntA**	**EntF**	**EntB’**	**EntB**	**EntE**	**EntC**
S. indicatrix WS40	84	83	85	93	93	82
S. lactitubi M15	84	83	86	92	93	83
S. pavanii CCUG 59972T	91	95	92	97	96	91
S. bentonitica DSM 103927	75	69	70	81	81	65
S. rhizophila IS26	74	71	68	82	81	65
S. riyadhensis e-p10	92	93	- **	97	96	91
S. muris DSM 28631	94	95	- **	98	96	90
**AVERAGE**	85	84	80	91	91	81

** Denotes the absence of any potential match based on the *E* value threshold of 0.05.

Table S4 Closest homologs of *S. maltophilia* EntB in other bacteria

Species (number of other species in genus with similar homology)	Annotation	Accession Number	E value	% Identity
Pseudomonas species*	Putative isochorismatase	HCL42795.1	4.00E-146	98.57
Variovorax species AB1 2024	Putative isochorismatase	WP_339449635.1	1.00E-117	81.99
Uliginosibacterium species H1	Putative isochorismatase	WP_327604145.1	3.00E-117	81.99
Streptomyces species II-2-2-2	Putative isochorismatase	XGB23282.1	3.00E-117	81.52
Luteimonas fraxinea (28)	Putative isochorismatase	WP_269781329.1	9.00E-114	78.1
Xanthomonas chitinilytica (2)	Putative isochorismatase	WP_265129199.1	1.00E-107	82.86
Candidatus *Dactylopiibacterium carminicum* (2)	Putative isochorismatase	WP_095523247.1	2.00E-106	77.25
Pseudothauera nasutitermitis	Putative isochorismatase	WP_136348239.1	2.00E-106	76.67
Salinicola rhizosphaerae	Putative isochorismatase	WP_189445479.1	7.00E-106	73.46
Salinicola acroporae	Putative isochorismatase	WP_110717098.1	2.00E-104	74.41
Candidatus *Oceanisphaera merdipullorum*	Putative isochorismatase	MBU3825022.1	3.00E-104	74.29
Halomonas garicola	Putative isochorismatase	WP_311954754.1	1.00E-102	73.46
Pseudomonas species*	Putative isochorismatase	MDY0206470.1	5.00E-99	72.38
Oceanimonas baumannii (5)	Putative isochorismatase	WP_228212323.1	2.00E-98	77.14
Oceanisphaera marina	Putative isochorismatase	WP_188628989.1	2.00E-96	73.33
Neomegalonema species*	Putative isochorismatase	WP_300149227.1	4.00E-96	69.81
Xanthomonas species XNM01	Putative isochorismatase	WP_192283326.1	4.00E-96	68.9
Chromohalobacter canadensis	Putative isochorismatase	WP_277810074.1	5.00E-96	74.41
Roseomonas gilardii (12)	Putative isochorismatase	WP_027280802.1	3.00E-95	69.86
Rhizobium species YJ-22	Putative isochorismatase	WP_277918899.1	1.00E-94	67.77
Pseudoroseomonas wenyumeiae (4)	Putative isochorismatase	MFC7553377.1	3.00E-94	68.42
Oceanisphaera avium	Putative isochorismatase	WP_086963543.1	7.00E-94	69.52
Neomegalonema perideroedes	Putative isochorismatase	WP_018632569.1	1.00E-93	68.4
Ancylobacter species* (1)	Putative isochorismatase	HEY9216060.1	1.00E-93	68.27
Mesorhizobium species YR577 (1)	Putative isochorismatase	WP_091918282.1	1.00E-93	67.8
Aquamicrobium segne	Putative isochorismatase	WP_378230451.1	3.00E-93	66.19
Comamonas serinivorans	Putative isochorismatase	WP_087282481.1	6.00E-93	68.12
Ectorhizobium quercum	Putative isochorismatase	WP_306409744.1	2.00E-92	67.77
Blastochloris viridis	Putative isochorismatase	WP_055038396.1	4.00E-92	66.99
Puniceibacterium sediminis	Putative isochorismatase	WP_089273683.1	5.00E-92	68.1
Paracoccus species* (2)	Putative isochorismatase	WP_374427208.1	6.00E-92	68.57

Geminicoccus species*	Putative isochorismatase	HMR30562.1	7.00E-92	68.1
Streptomyces species ATCC 700974	DhbB isochorismatase	CBA63658.1	1.00E-62	45.7

*NCBI did not specify below the species level.

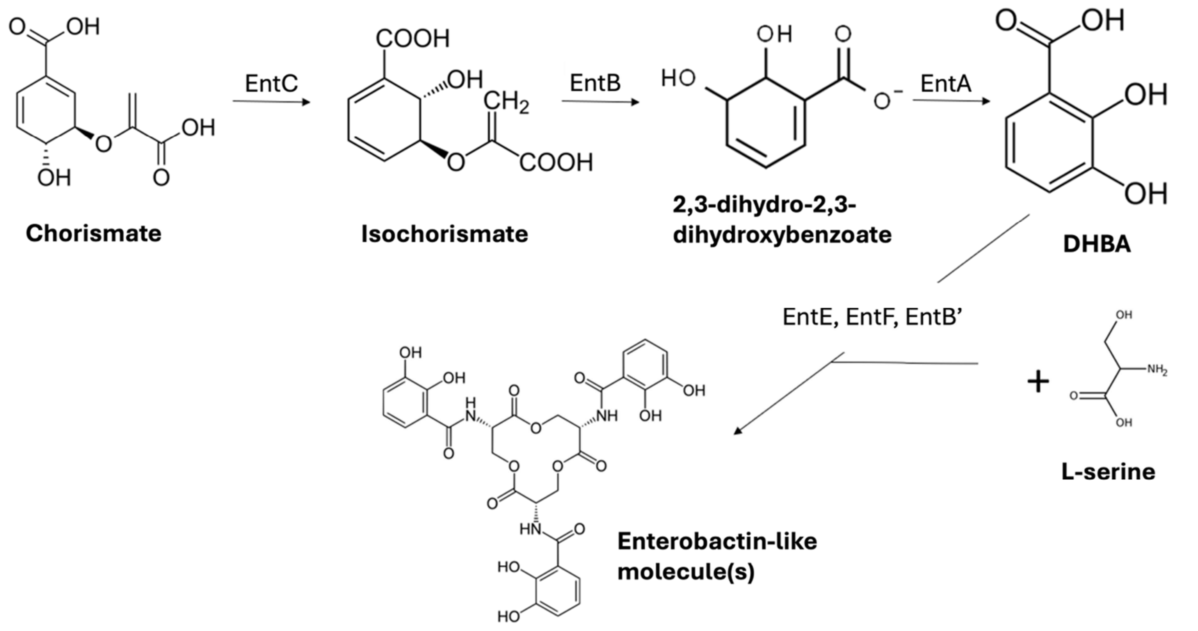

Figure S1 Proposed *S. maltophilia* siderophore biosynthesis. Shown are the proposed reactions mediated by EntC, EntB, EntA, EntE, EntF, and EntB', which are encoded by the *entCEBB'FA* locus. Deviations from *E. coli* enterobactin biosynthesis include the separation of isochorismatase and ArCP domains into two proteins, EntB and EntB'. Additionally, the *S. maltophilia* equivalent to *E. coli* EntD is unknown.

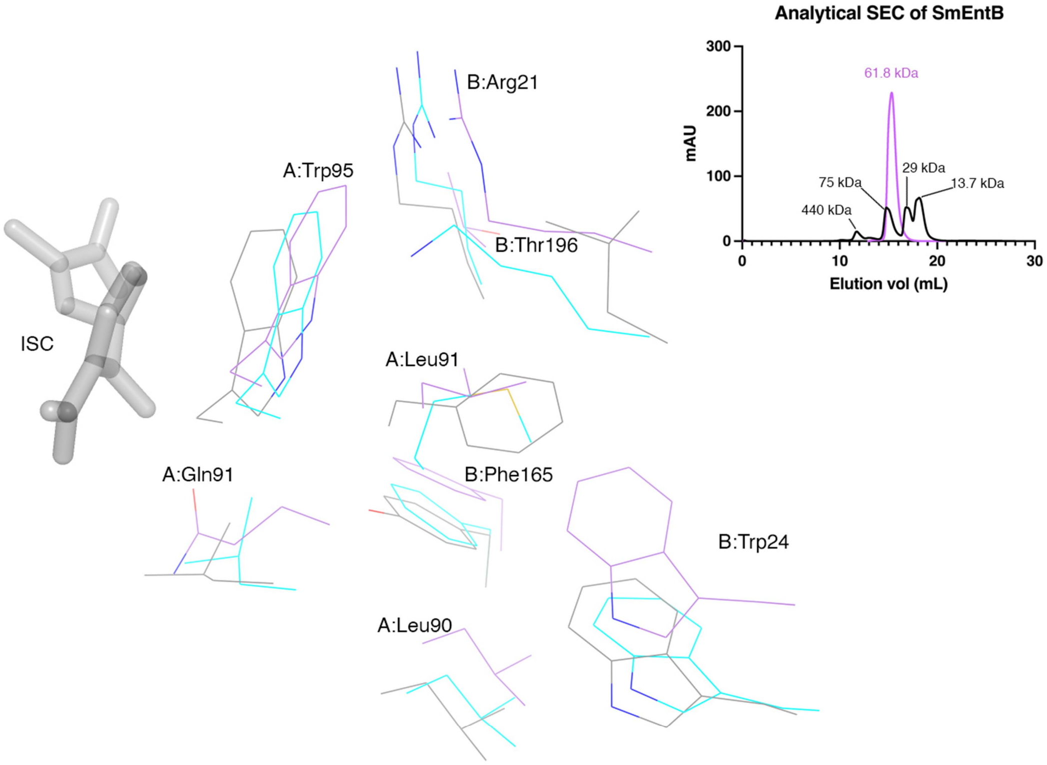

Figure S2 Comparison of EntB dimer interface residues. An overlay of the *Sm*EntB crystallographic dimer with that of *Ec*EntB (cyan carbon backbone) and *Pa*PhzD (gray carbon backbone). The side chains of dimer interface residues, as described in (Drake *et al.*, 2006), are shown as lines. Residue labeling is based on *Sm*EntB numbering. The ISC molecule from *Pa*PhzD structure is labeled and shown as gray sticks. The inset graph shows the analytical SEC curve of *Sm*EntB overlaid with that of protein weight standards used to estimate the solution size of *Sm*EntB.

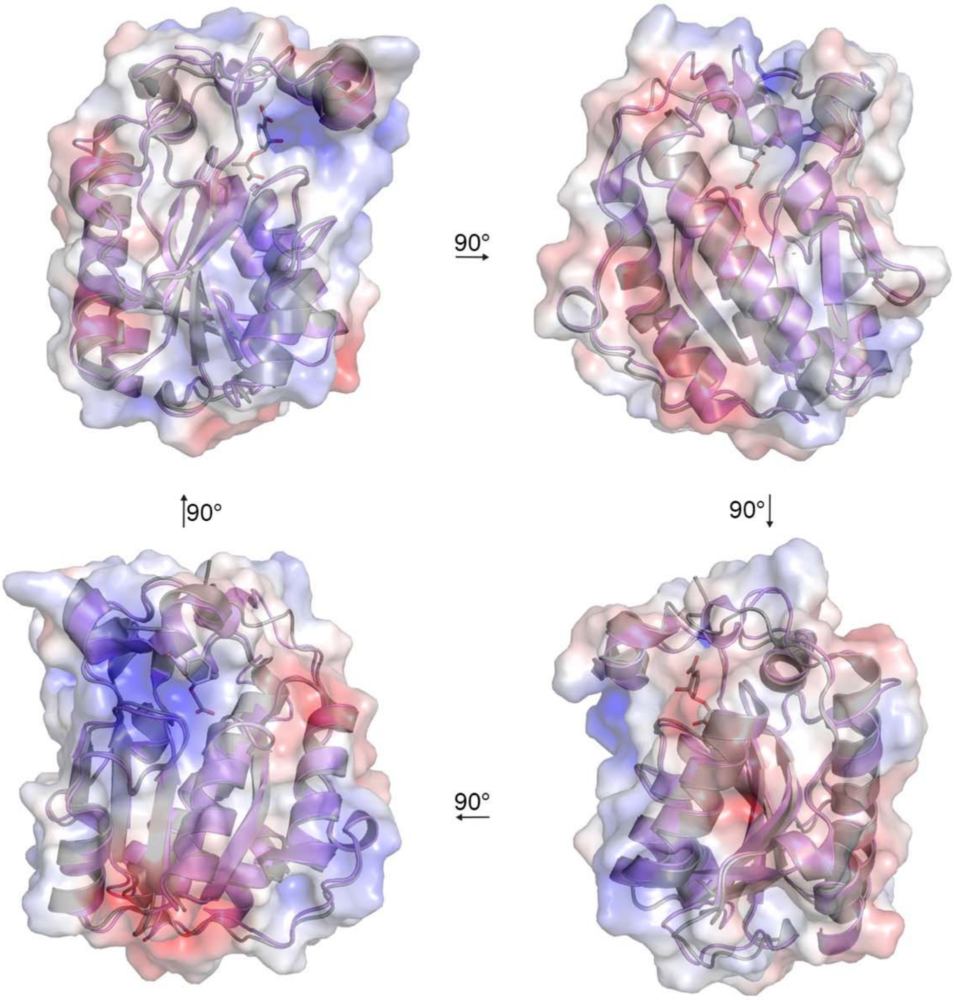

Figure S3 Electrostatic surface projection of *Sm*EntB overlaid with the *Pa*PhzD structure. The carbon backbones of *Sm*EntB (PDB 7lj6 lilac) and *Pa*PhzD (1nf8 grey) structures were overlaid and are shown as cartoons. Isochorismate from the PaPhzD structure is shown as sticks with nitrogen atoms in blue and oxygen atoms in red. The electrostatic surface of SmEntB was predicted using APBS in PyMol. Four views of the globular structure are provided.

Reference

Drake, E. J., Nicolai, D. A. & Gulick, A. M. (2006). *Chem. Biol.* **13**, 409-419.
